# High-Throughput Genome-Wide Genotyping To Optimize the Use of Natural Genetic Resources in the Grassland Species Perennial Ryegrass (*Lolium perenne* L.)

**DOI:** 10.1534/g3.120.401491

**Published:** 2020-07-21

**Authors:** Thomas Keep, Jean-Paul Sampoux, José Luis Blanco-Pastor, Klaus J. Dehmer, Matthew J. Hegarty, Thomas Ledauphin, Isabelle Litrico, Hilde Muylle, Isabel Roldán-Ruiz, Anna M. Roschanski, Tom Ruttink, Fabien Surault, Evelin Willner, Philippe Barre

**Affiliations:** * INRAE, Centre Nouvelle-Aquitaine-Poitiers, UR4 (UR P3F), F-86600 Lusignan, France; † Leibniz Institute of Plant Genetics and Crop Plant Research (IPK), Inselstr. 9, 23999 Malchow/Poel, Germany; ‡ IBERS-Aberystwyth University, Plas Goggerdan, Aberystwyth, Ceredigion, United Kingdom; § Flanders Research Institute for Agriculture, Fisheries and Food (ILVO) - Plant Sciences Unit, Caritasstraat 39, 9090 Melle, Belgium

**Keywords:** natural diversity, genebank, association study, genomic prediction, forage species, GWAS, GenPred, Shared data resources

## Abstract

The natural genetic diversity of agricultural species is an essential genetic resource for breeding programs aiming to improve their ecosystem and production services. A large natural ecotype diversity is usually available for most grassland species. This could be used to recombine natural climatic adaptations and agronomic value to create improved populations of grassland species adapted to future regional climates. However describing natural genetic resources can be long and costly. Molecular markers may provide useful information to help this task. This opportunity was investigated for *Lolium perenne* L., using a set of 385 accessions from the natural diversity of this species collected right across Europe and provided by genebanks of several countries. For each of these populations, genotyping provided the allele frequencies of 189,781 SNP markers. GWAS were implemented for over 30 agronomic and/or putatively adaptive traits recorded in three climatically contrasted locations (France, Belgium, Germany). Significant associations were detected for hundreds of markers despite a strong confounding effect of the genetic background; most of them pertained to phenology traits. It is likely that genetic variability in these traits has had an important contribution to environmental adaptation and ecotype differentiation. Genomic prediction models calibrated using natural diversity were found to be highly effective to describe natural populations for almost all traits as well as commercial synthetic populations for some important traits such as disease resistance, spring growth or phenological traits. These results will certainly be valuable information to help the use of natural genetic resources of other species.

The impacts of climate change are already becoming visible, notably with an increase in the frequency, length and intensity of seasonal droughts (Overpeck 2013). These climatic stresses make plant stands survival a growing challenge (Allen *et al.* 2010; Griffin and Hoffmann 2012; Pivovaroff *et al.* 2016; Volaire 2018). The *in-situ* genetic diversity of a species present at a local scale may not be sufficient to provide adaptation to possibly rapid changes in climatic conditions and this could result in species extinction in some locations (Vitt *et al.* 2010). The threatened natural genetic diversity can be important for food production (Jarvis *et al.* 2008), notably in the case of Crop Wild Relatives (CWR) which are used as sources of genetic diversity in crop breeding and can help agriculture face various challenges such as climate change (Hajjar and Hodgkin 2007; Breithaupt 2008). Furthermore, losses of some species may be detrimental to the capacity of natural and semi-natural ecosystems such as grasslands and forests to provide economic and ecosystem services via biomass production, carbon sequestration, preservation of water quality and biodiversity (Reheul *et al.* 2010). Major changes in species composition could ultimately lead to drastic changes in the land use, possibly including conversions of grasslands to arable land (Dengler *et al.* 2014).

The whole diversity of a species present over a wide range of environments could be sufficient to cope with changing local climates. Collecting and describing the natural diversity of plant species over a wide environmental range is thus an essential first step to the process of understanding adaptive strategies and finding original genetic diversity. Such diversity could be used to provide or to improve adaptation in stressful environments. However, characterizing and evaluating an extended range of diversity within a species is time consuming and expensive and so is seldom undertaken. Choosing a sub-set of accessions to be carefully characterized appears essential. For some species, notably those of agricultural importance such as CWR, *ex-situ* collections of natural diversity have been collected and are maintained in genebanks. Current collections often do not reflect full intra-specific diversity (Castañeda-Álvarez *et al.* 2016) but are an accessible sample of the diversity that can be readily described and analyzed. It has been proposed that phenotyping data of genebank material could be efficiently completed by genotyping data from next-generation sequencing methods to describe and valorise wide natural or domesticated genetic resources (Ford-Lloyd *et al.* 2011; Thorwarth *et al.* 2018).

Molecular markers have already proved to be efficient in plant species for revealing patterns of genetic diversity structure (El Bakkali *et al.* 2013), creating core collections (van Hintum *et al.* 2000), identifying major effect QTL through Genome Wide Association Studies (GWAS) (Zhao *et al.* 2011) and Genomic Prediction (GP) models (Meuwissen *et al.* 2001; Kooke *et al.* 2016). Here, we intented to evaluate the usefulness of molecular markers in characterizing the overall diversity of perennial ryegrass (*Lolium perenne* L.) and in optimizing their use for mining genetic resources.

Perennial ryegrass is an example of highly valuable grassland species spread over a wide range of environments throughout Europe and the Mediterranean area but locally threatened by changing climate (Wilkins 1991; Blackmore *et al.* 2016; Blanco-Pastor *et al.* 2019). Thus, wide natural genetic resources exist in this species and can be exploited to breed genetic material with novel trait combinations adapted to new climatic constraints and meeting agricultural needs. The advances in genotyping technologies for perennial ryegrass, with a recently sequenced genome (Byrne *et al.* 2015) and the affordable Genotyping-By-Sequencing technique (Elshire *et al.* 2011), make possible to describe molecular diversity over the whole genome for a large set of populations (Blanco-Pastor *et al.* 2019). Association studies have already been undertaken using genetic and phenotypic data recorded at individual as well as at population level in perennial ryegrass. However these studies were addressing genetic material from breeding programs and were focused on some specific agronomic targets such as phenological traits, yield, disease resistance and quality traits (Skøt *et al.* 2007; Fè *et al.* 2015a; Arojju *et al.* 2016). These studies revealed major effect QTL explaining substantial proportions of phenotypic variability, notably for heading date (Arojju *et al.* 2016). Some of these QTL were found close to, or within, genes presenting homologs with known functions in other species (Fè *et al.* 2015a). GWAS however suffer from drawbacks that limit the capacity of this approach to provide an exhaustive overview of genomic polymorphisms involved in phenotypic trait variation. For example, phenotypic variation is often confounded to a certain extent with genetic structure in the set of genotypes under study (Zhao *et al.* 2011). This issue is especially a concern in studies using wide samples of diversity rather than populations of recombinant genotypes.

GP consists of predicting breeding values from the cumulated information of many markers spread all across the genome. The interest of GP has already been demonstrated to assess the genetic value of natural or domesticated materials maintained in genebanks for wheat (Crossa *et al.* 2016), sorghum (Yu *et al.* 2016), cauliflower (Thorwarth *et al.* 2018), pea (Burstin *et al.* 2015), soybean (Peixoto *et al.* 2017) and Arabidopsis (Kooke *et al.* 2016).

In perennial ryegrass, the implementation of GP resulted in a range of predictive ability values depending on different factors such as the heritability of the trait, the size of the calibration set, the marker density and the relatedness between the calibration set and the evaluated genetic material (Fè *et al.* 2015a; Grinberg *et al.* 2016; Faville *et al.* 2018; Pembleton *et al.* 2018; Cericola *et al.* 2018). GP has been used in this species to predict i) the phenotypes of individuals evaluated as spaced plants (Grinberg *et al.* 2016), ii) the sward performances of progeny families, including half sib families (Faville *et al.* 2018) and full sib families (Fè *et al.* 2015a) and iii) the sward performances of synthetic cultivars (Pembleton *et al.* 2018). All these studies only addressed elite genetic material from breeding programs.

By contrast, the present study is based on a set of 385 genebank accessions representing natural populations of perennial ryegrass from almost the whole region of natural expansion of the species, *i.e.*, Europe and the Near-East. These accessions were phenotyped for agronomic and putatively adaptive traits at three climatically contrasted trial locations (France, Belgium and Germany) and genotyped using Genome-Wide Allele Frequency Fingerprinting with 189,781 SNP markers. The objectives were i) to evaluate the natural variability of agronomic and putatively adaptive phenotypic traits, ii) to identify SNP markers linked to trait variation (GWAS) that could be afterward used to screen larger diversity by targeted sequencing, iii) to estimate the potential of GP models to accurately predict the genetic values of natural populations and to efficiently screen very large collections of *in-situ* material, iv) to test whether such GP models could be optimized notably by using a limited number of representative accessions in the calibration set (which is equivalent to identifying an optimized genebank core collection).

## Material and methods

### Plant material

**Figure 1:**
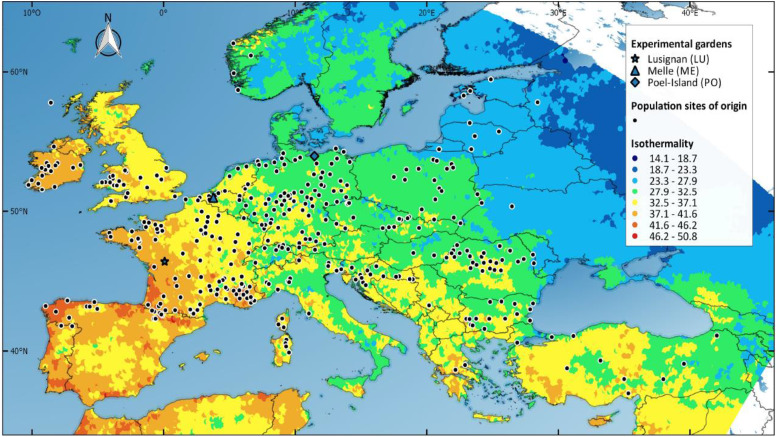
Spatial distribution of sites of origin of the 385 perennial ryegrass natural populations in study and of locations of the three common gardens in which populations were phenotyped. The 1989-2010 norm of isothermality, *i.e.*, mean temperature diurnal range over annual temperature range (WorldClim bioclimatic derived variable *bio3*) is displayed as map background.

This study used 385 perennial ryegrass accessions from natural populations provided by European genebanks as well as 32 cultivars. The set of natural populations was chosen as to represent the intraspecific diversity across Europe and the Near-East as presented in Figure 1. Sites and dates of collect are reported in Table S1. Natural populations from *L. perenne* and other *Lolium* taxa were collected by scientists and plant breeders from a number of agronomic research institutes in Europe and the USDA between years 1960 and 2013. The sampling, regeneration and conservation protocols for the seed lots of collected populations are described in Table S1. Seed lots were regenerated and increased once soon after collection and then once or twice again (each 15 years) for the oldest accessions in field facilities available to genebanks. Regeneration of a population is performed by the panmictic intercross of a number of plants (50 to 200) isolated from pollen flow of other plants of the species. Balanced seed bulks harvested on such intercrossed plants can be considered as a mixture of half-sib family genotypes. The taxonomy (*L. perenne* L) and the caryotype (diploid) of populations was checked from plant material grown in experimental common gardens (see latter paragraph ‘Experimental design’). Seeds samples of cultivars were extracted from seed lots produced for seed trade by plant breeders from three generations of intercross in panmixia from 8 to 30 selected founder genotypes. These seed samples can thus also be considered as mixtures of half-sib family genotypes. The list of cultivars with their origin is reported in Table S2.

### Genotyping

For the needs of the genotyping, 300 seeds were drawn from the most recent bulk of seeds of each of the selected natural populations and from the available seed lot of commercial cultivars to grow 300 seedlings for a common DNA extraction. Genotyping was performed at the population level by estimating the allele frequencies of nuclear genome SNP markers through sequencing the pools of 300 bulked DNAs. Two methods were used to sequence a reduced, yet consistent fraction of the genome: 1) Genotyping by Sequencing (GBS) described by Elshire *et al.* (2011) in order to provide genome-wide coverage and 2) highly multiplexed amplicon sequencing (Hi-Plex) developed by Floodlight Genomics LLC (Knoxville, TN, USA, https://floodlightgenomics.com) to target candidate genes putatively involved in aerial morphogenesis (Veeckman *et al.* 2019).

GBS with the *Pst*I restriction enzyme was performed on DNA extracted from pooled leaf material (Byrne *et al.* 2013). The experimental and bioinformatics methods were described in detail by Blanco-Pastor *et al.* (2019) who used the same genetic material and genotyping data. A minimum read depth per locus of 30 was targeted and the final average read depth across all loci equalled 150.

For the Hi-Plex sequencing, primers were designed with Primer3 (Untergasser *et al.* 2012) in candidate genes using previous knowledge on their allele sequence polymorphism (Barre *et al.* 2014; Veeckman *et al.* 2019). A set of 185 amplicons of 80-140 bp were sequenced (Table S3).

Only bi-allelic SNPs from the GBS and the Hi-Plex sequencing were kept. Allele frequencies were estimated using SNAPE-pooled (Raineri *et al.* 2012). A filter was applied so that the minor allele frequency of each SNP marker was higher than 5% in at least 10 populations. Eventually, variant calling delivered population allele frequencies for 189,781 SNP markers distributed over 10,335 scaffolds of the perennial ryegrass reference genome sequence (Byrne *et al.* 2015), including 524 SNP markers in 42 candidate gene loci from Hi-Plex. The percentage of missing data per SNP marker was on average 8% (range 0–33%). Each missing data were replaced by the mean allele frequency across all populations.

The raw genetic data (sequences) reported in this study are available in the NCBI Short Read Archive (SRA) database through accession SRP136600. The allele frequencies per population are available in Table S4.

### Experimental design

The 385 perennial ryegrass populations as well as the 32 cultivars were sown in experimental common gardens in three locations: Poel Island (PO) in Germany (53.990 N, 11.468 E) on the 8^th^ of April 2015, Melle (ME) in Belgium (50.976 N, 3.780 E) on the 2^nd^ of October 2015 and Lusignan (LU) in France (46.402 N, 0.082 E) on the 9^th^ of April 2015. They were sown in three complete blocks in each location. Micro-swards of 1 m^2^ were sown with 2, 4 or 6 g of seeds according to whether the previously checked germination rate was higher than 80%, between 80% and 60%, or smaller than 60%, respectively. An amount of 2 g m^-2^ seeds of good germination quality (> 80%) is the seed density commonly used to sow dense perennial ryegrass meadows for forage usage. Trials were monitored until end of 2017 in PO and ME and until end of 2018 in LU. Micro-swards were regularly cut at 7 cm above ground surface. Cutting dates were 16/06/15, 06/08/15, 04/09/15, 12/10/15, 04/03/16, 01/06/16, 13/07/16, 31/08/16, 26/10/16, 10/03/17, 07/06/17, 19/07/17, 01/09/17, 13/10/17 at PO; 13/05/16, 08/07/16, 29/08/16, 13/10/16, 18/04/17, 31/05/17, 13/07/17, 24/08/17, 04/10/17 at ME and 30/06/15, 03/09/15, 30/10/15, 04/02/16, 08/06/16, 26/07/16, 01/02/17, 13/06/17, 07/09/17, 07/06/18, 27/08/18 at LU. Anti-dicotyledon herbicide was applied once in 2015 in each location and a second time in 2016 or 2017 according to location. Nitrogen fertilization was applied with 60 kg N ha^-1^ two months after sowing and after each aerial biomass cut and with 80 kg N ha^-1^ after each winter (2015-2016 and 2016-2017) at start of spring growth.

Weather conditions experienced at each trial location are displayed per season of each year in Table S5. At LU, drought stress was severe during summers of 2016 and 2017. During these summer periods, the average soil water content fell below 20% of the soil water content at field capacity. At PO, no signs of drought stress were detected but the winter periods were colder (mean temperature below 3°) than at the two other locations, especially at the end of the 2015-2016 winter period. At ME, periods of moderate drought stress occurred during summer and autumn, notably during the summer of 2017 when soil water content fell below 27% of the soil water content at field capacity.

### Recorded phenotypic traits

Scores or measurements of phenotypic traits were recorded at the level of 1m^2^ micro-swards. A large set of traits was assessed in order to evaluate both the agronomic and the adaptive capacity of the vast diversity of perennial ryegrass populations. The traits recorded are briefly presented in Table 1 and described in detail in File S1.

**Table 1:** Brief presentation of the traits

Trait family	Trait description	Trait name*^a^*
Vigor after sowing	Days from sowing to emergence	DES_po15
	Vigor after sowing	VAS-lu15, VAS_me15, VAS_po15
	Regularity after sowing	RAS_lu15, RAS_me15, RAS_po15
Morphology of plants and sward density	Leaf lamina width	LMW_po16, LMW_me16, LMW_lu17
	Growth habit	GRH_avg
	Sward density	DVG_04_lu17
Phenology	Percentage of plants heading in first year	HFY_lu15, HFY_po15
	Spike emergence (heading) date in GDD*^b^*	HEA_lu16, HEA_po16, HEA_lu17, HEA_po17
	Aftermath heading (Second wave of fertile elongating stems after the first spring wave has been cut)	AHD_lu16, AHD_lu17, AHD_me16, AHD_me17, AHD_po16, AHD_po17
Investment in sexual reproduction	Density of elongated fertile stems*^c^*	DST_lu17, DST_po17
	Straw height	HST_lu17
	Spike length	LSP_lu17
	Spikelet length	LSL_lu17
	Spikelet count	NSL_lu17
Dynamics of vegetative spring growth	Canopy height at 300 GDD*^b^* after the start of spring growth*^c^*	CHs300_lu16, CHs300_lu17, CHs300_po16, CHs300_po17, CHs300_me16, CHs300_me17
	Canopy height at 500 GDD*^b^* after the start of spring growth*^c^*	CHs500_lu16, CHs500_lu17, CHs500_po16, CHs500_po17, CHs500_me16, CHs500_me17
	Canopy height at 300 GDD*^b^* before spike emergence (heading) date*^c^*	CH300h_lu16, CH300h_lu17, CH300h_po16, CH300h_po17
	Canopy height at 400 GDD*^b^* before spike emergence (heading) date*^c^*	CH400h_lu16, CH400h_lu17, CH400h_po16, CH400h_po17
Summer and autumn growth	Summer maximum canopy height	SMH_lu16, SMH_me16, SMH_me17, SMH_po17
	Summer growth rate	SGR_lu16, SGR_me16, SGR_me17, SGR_po17
	Autumn maximum canopy height	AMH_me17, AMH_po17
	Autumn growth rate	AGR_me17, AGR_po17
Dynamics of regrowth after cutting	Vigor after cutting	VAC_lu16, VAC_lu17, VAC_po17
Abiotic stresses	Drought stress symptoms	DRO_lu16, DRO_po16
	Winter damage	WID_po16, WID_po17
Biotic stresses - Disease damages	Helmintosporium (Dreschlera siccans) susceptibility	DHE_01_lu16, DHE_07_lu16, DHE_04_lu17
	Black rust (Puccinia graminis) susceptibility	DRB_lu1516
	Susceptibility to indeterminate diseases	DIS_lu15, DIS_lu16, DIS_lu17, DIS_me16, DIS_me17, DIS_po15, DIS_po17
Dynamics of persistency over successive trial years	Persistency throughout summer	SCD_su15_lu, SCD_su16_lu, SCD_su17_lu, SCD_su17_me
	Persistency throughout winter	SCD_wi1516_lu, SCD_wi1617_lu, SCD_wi1718_lu, SCD_wi1617_me, SCD_wi1516_po, SCD_wi1617_po
	Persistency throughout the trial duration	SCD_15to18_lu, SCD_15to17_po, SCD_16to17_me
Biochemistry of aerial biomass	Lignin content	ADL_04_me17, ADL_10_me17
	Acid-Detergent-Fiber content	ADF_04_me17, ADF_10_me17
	Neutral-Detergent-Fiber content	NDF_04_me17, NDF_10_me17
	Crude protein content	PRT_04_me17, PRT_10_me17
	Water-soluble-carbohydrate content	WSC_04_me17, WSC_10_me17
	Neutral-Detergent-Fiber degradability	DNDF_04_me17, DNDF_10_me17
	Organic matter digestibility	OMD_04_me17, OMD_10_me17
	Nitrogen content of sunlit leaf lamina	NLI_lu16
	Isotopic composition of ^13^C (δ^13^C)	13C_lu16

a After _, the trait name is suffixed by the location (PO for Poel, LU for Lusignan and ME for Melle) and the year (avg for average, and for example, 15-18 for 2015-2018)

b dates were converted into growing degree days (GDD) with a base temperature of 0°C starting from the first day when daily minimum temperature and incident shortwave global radiation do not fall anymore below 0°C and 60 W m-2, respectively (*i.e.*, from the start of vegetative spring growth)

c in addition new variables indicated by *res* at the beginning of the variable name were obtained by removing the effect of average heading date (HEA_avg)

### Computation of mean values per population and of indicators analogous to heritabilities

Models of analyze of variance (ANOVA) were used to check the accuracy of the raw data and the significance of the population effect and to compute adjusted means per population. Analyses of variance were performed using the R functions “*lm*” and “*Anova*” of the R (R Core Team 2018) “*car*” library.

For each location-trait combination, the following fixed effect model was used:

$${\text{Y}}_{\text{ij}}=\text{µ}+g_{i}+b_{j}+E_{\mathrm{ij}}\text{(model 1)}$$

where Y_ij_ is the observed value of population *i* in the complete block *j*, g_i_ is the (genetic) effect of population *i*, b_j_ is the effect of the complete block *j* and E_ij_ is the residual effect of the model.

F statistics of the population effect were highly significant (*pvalue* < 0.005) for all combinations of traits, locations and record dates (can be different periods of a same year), in agreement with satisfactory accuracy of collected raw data. Adjusted means per location and date were computed using the “*emmeans*” function of the “*emmeans” R* library. These adjusted means were used as population values for downstream analyses.

For traits for which it was relevant, an analysis of variance was also performed across tested combinations of locations and dates of record (*i.e.*, environments) using the following model:

$${\text{Y}}_{\text{ijr}}=\text{µ}+g_{i}+{\mathrm{env}}_{j}+\text{g}\times {\mathrm{env}}_{\mathrm{ij}}+{\text{b/env}}_{\text{jr}}+{\text{E}}_{\text{ijr}}\text{(model 2)}$$

where Y_ijr_ is the observed value of population *i* within the complete block *r* of environment *j*, g_i_ is the (genetic) effect of population *i*, env_j_ is the effect of environment *j*, g × env_ij_ is the interaction between population *i* and environment *j*, b/env_jr_ is the effect of complete block *r* nested within environment *j* and E_ijr_ is the residual of the model.

Analyzing model 2 as a mixed model with g_i_, and b/env_jr_ as a fixed effects and env_j_ and g × env_ij_ as random effects resulted in highly significant F statistics (*pvalue* < 0.005) of the g_i_ effect for all traits recorded in more than one location × record date combination. For these traits, it was thus relevant to use population means across environments for downstream analyses. These were computed using the “*emmeans*” function. The mean of a trait across environments was identified in downstream analyses by adding to the name of the trait the suffix *avg* (*e.g.*, ***VAS_avg***, ***RAS_avg***, ***LMW_avg***, ***GRH_avg***, ***HFY_avg***, ***HEA_avg***, ***AHD_avg***, ***VAC_avg***, ***DRO_avg***, ***DIS_avg*, *ADF_avg*, *ADL_avg*, *NDF_avg*, *OMD_avg*, *WSC_avg*, *DNDF_avg*,** and ***PRT_avg***).

All the adjusted means of phenotypic traits from models 1 and 2 are available in Table S6.

For each phenotypic variable, H^2^ indicators analogous to broad-sense heritability were computed by setting the g_i_ effect as random in models (1) and (2) and using only the natural population data.

Then for traits recorded in only one environment:

$${\text{H}}^{2}=\sigma_{\text{g}}^{2}/\left(\sigma_{\text{g}}^{2}+\text{1/R}\sigma_{\text{e}}^{2}\right)$$

where σ^2^_g_ is the variance of the population (g_i_) effect, σ^2^_e_ is the residual effect of the ANOVA and R is the number of replicates of populations per environment.

And for traits recorded in several environments:

$${\text{H}}^{2}=\sigma_{\text{g}}^{2}/(\sigma_{\text{g}}^{2}+\text{1/J}\sigma_{\text{ge}}^{2}+\text{1/}\left(\text{JR}\right)\sigma_{\text{e}}^{2})$$

where σ^2^_ge_ is the variance of the g × env_ij_ interaction effect and J is the number of environments.

### Spatial analyses of trait values and SNP allele frequencies

The Moran’s index (I) is an indicator of spatial autocorrelation for a variable distributed across space. It was used for phenotypic trait values. It varies between -1 (perfect dispersion) and 1 (perfect autocorrelation). Values close to 0 indicate that the spatial distribution of the variable is perfectly random. Spatial distances between sites of origin of populations were calculated from their geographical coordinates using the “*distm*” function of the R “*geosphere*” package. Moran indices were then calculated using the “*Moran.I*” function of the “*ape*” R package.

To investigate the correlation between different kinds (genetic, phenotypic or geographic) of distance matrices, Mantel correlations were computed. The Mantel correlation between two distance matrices was calculated using the “*mantel*” function of the R “*ecodist*” package with 500 permutations to test the significance level of the correlation. Pairwise distances could either be geographic distances in meters, genetic Euclidian distances or phenotypic Euclidian distances. Such analyses were notably used to investigate phenotypic and genetic isolation by geographical distance.

### Kinship, genetic structure and correlation between markers

Kinship between populations was estimated using a genomic relationship matrix (Endelman and Jannink 2012). SNP data were assembled into a n×p matrix X_n*p_ (*n* populations and *p* markers) giving the alternative allele frequency of each SNP marker in each population. The genomic relationship matrix (G) was calculated using the following formula:

$$\text{G}=MM^{\prime }/\text{K}$$

where M*_np_ is the X*_np_ matrix in which each column is centered by the average alternative allele frequency and K is a scaling parameter representing the sum of genetic variance (Cericola *et al.* 2018) quantified as $0.5\sum_{j=1}^{p}\bar{X_{j}}(1-\bar{X_{j}})$ with $\bar{X_{j}}=\frac{1}{n}\overset{n}{\underset{i=1}{\sum }}X_{ij}$.

The genetic structure of the set of studied populations was previously analyzed by Blanco-Pastor *et al.* (2019) who identified seven clusters displaying some degree of admixture and a weak level of differentiation between spatially close clusters. Furthermore, the correlation between pairwise geographic distances and pairwise genetic Euclidian distances calculated using the allele frequencies of all available SNP markers is of 0.44. As such, the genetic differentiation seems to follow a relatively continuous pattern suggesting a geographic isolation by distance. Because this study used population allele frequencies and not allele content of individuals, it was not possible to calculate linkage disequilibrium (LD) between SNP markers. Squared correlations between allele frequencies of pairs of SNP were instead calculated. Correlation decay with increasing base pair (bp) distance was computed for SNP markers belonging to a same scaffold.

### Association between markers and traits

The “*GWAS*” function of the R “*rrBLUP*” package was used to perform the GWAS analyses. The analyses were performed either without taking into account kinship or structure, taking into account only structure (seven clusters from Blanco-Pastor *et al.* 2019) or only kinship (G), or taking into account both structure and kinship.P-values were adjusted into q-values using a false discovery rate (FDR) controlling procedure (Benjamini and Hochberg 1995). For each trait, SNP markers with a q-value lower than 10% were considered significant. The percentage of trait variance explained by the SNP and the alternative allele fixed effect estimate were computed with kinship taken into account in the GWAS model. Furthermore, for each significant SNP, a linear regression between the population trait value and the SNP alternative allele frequency was used to estimate the alternative allele effect (slope) and the phenotypic variance explained (R^2^) without accounting for kinship.

A forward stepwise multiple regression model (starting with the most significant SNP and ending with least significant detected SNP) was finally implemented using the “*step*” R function to predict the phenotypic trait from a set of non-redundant significant SNPs. From this regression model, the total phenotypic variance explained by all non-redundant significant SNPs was assessed.

### Genomic predictions

#### General model:

The RR-BLUP model (Meuwissen *et al.* 2001), implemented in the “*mixed.solve*” function of the “*rrBLUP*” R package, was used for GP. Either all available SNP markers were used or different sets of SNP markers were used according to preliminary GWAS results. Model parameters were estimated using REML.

The RR-BLUP model was chosen because it has been proven to perform well for GP and it is generally not surpassed by other methods such as various Bayesian models or random forest computational approaches (Bao *et al.* 2015; Faville *et al.* 2018; Pembleton *et al.* 2018). For each trait, the model was evaluated by cross validation using 100 random sub-sampling of 50 populations that were removed for model calibration and then predicted. The average correlation over the 100 sub-samples between observed phenotypic values and predicted values was used as an estimate of predictive ability.

We also investigated for different traits whether GP models calibrated using all the natural populations could predict the average phenotypic value of cultivars. This was assessed by computing the correlation between observed and predicted values of cultivars.

#### Optimization of the calibration set for genomic predictions:

Phenotyping is a major bottleneck of the assessment of wide collections of genetic resources. Therefore, it is worthwhile to use the smallest possible calibration set to set up GP models. We tested five different procedures to select the calibration set and compared the predictive abilities provided by the resulting GP models for all traits. For each procedure, different calibration set sizes (k) were tested.

For the first procedure, we computed an Euclidian genetic distance matrix between populations based on SNP allele frequencies using the R “*dist*” function. This distance matrix was used to cluster populations into k groups with the R “*hclust*” function. To create a calibration set of k populations, one population per group was selected. This population was the one which had the lowest average distance to the other populations in the group.

For the second procedure, we used a matrix of spatial distances (in meters) computed from spatial coordinates (longitude/latitude) of populations with the “*distm*” function of the “*geosphere*” R package. The process was the same as for the first procedure except that we used spatial distances instead of genetic distances.

For the third procedure, we used both spatial and genetic data. The process was similar to that of the first procedure except that the clustering method was a hierarchical genetic clustering with spatial constraints (Balfourier *et al.* 1998; Chavent *et al.* 2018). This clustering was performed using the Euclidian and spatial genetic distance matrices as input in the “*hclustgeo*” function of the “*ClustGeo*” R package with the mixing parameter set to 0.5.

For the fourth procedure, we used the mean of the coefficient of determination (CDmean) criterion (Rincent *et al.* 2012). A random calibration set of k populations was drawn to initiate the optimization and the CDmean was calculated. Then over 1000 iterations, one population was randomly exchanged between the calibration set and the pool of remaining populations and the CDmean criterion was recalculated. If the criterion was improved, the population exchange was accepted and otherwise rejected.

In order to calculate the performance of an optimized calibration set, the minimum (MinPA) and maximum (MaxPA) predictive abilities over 100 randomly selected calibration sets of populations with same sizes as the optimized set were computed. The performance of an optimized calibration set was then calculated as $\frac{\text{OptPA}-\text{MinPA}}{\text{MaxPA}-\text{MinPA}}$ with OptPA the predictive ability obtained with the optimized calibration set. For example, a performance of 0.5 indicates that the optimized predictive ability is the average between the minimum and the maximum predictive abilities of random samples.

#### Genomic predictions incorporating preliminary GWAS results:

The benefit of incorporating GWAS detected SNP markers to improve the efficiency of GP models was investigated for all traits. A calibration set of all minus 50 phenotyped populations was selected using the procedure 1 (optimization of genetic distances). The following steps were then implemented:

- A GWAS which accounted only for kinship was first implemented with the calibration set of populations. The SNP markers were ranked according to increasing p-values in GWAS analyses.
- SNP markers were added one by one as fixed effects in a multiple regression model predicting the phenotypic trait, starting from the SNP with smallest p-value. SNP markers were added only if the model was significantly improved (p-value of F-test below 5%) and the total number of included SNP markers could not exceed the number of populations in the calibration set so as to avoid overfitting. The SNP markers in the final model (*GWAS set*) were considered the most informative according to the GWAS.
- GP models (SNP markers as random effects) including from one to all SNP markers of the *GWAS set*, starting from the smallest p-value, were then calibrated and used to predict the genetic values of the populations in the validation set (50 populations).
- Randomly selected markers (not included in the *GWAS set*) were then included to the genomic prediction model one by one, up to 2000, in addition to all markers of the *GWAS set*.

The predictive ability could thus be displayed as a function of the number of SNP markers in the model. The minimum number of SNP markers needed to reach 95% of the highest predictive ability was determined. To evaluate the benefits of first adding SNP markers detected by GWAS on the calibration set, the same process was done but with the addition of random SNP markers from the start.

### Data availability

All the necessary data required to confirm the findings of this study is available in the supplemental material available at figshare: https://doi.org/10.25387/g3.12504422.

## Results

### Phenotypic diversity

Variation between populations was highly significant (*P* < 0.005) for all phenotypic variables. The H^2^ indicator within environment varied from 0.37 for summer growth rate at ME in 2017 (*SGR_me17*) to 0.98 for heading date at LU and PO in 2017 (*HEA_lu17* and *HEA_po17*) (Table S7). For a given trait, H^2^ was variable between trial sites, years and seasons (Table S7). The environment effect and the genotype × environment interaction were both significant (*P* < 0.05) for all traits observed over multiple environments. H^2^ computed from the ANOVA across environmental conditions (model 2) varied from 0.46 for vigor after sowing (*VAS*) scored in 2015 at LU, ME and PO to 0.99 for heading date (*HEA*) measured in 2016 and 2017 at LU and PO.

For all phenotypic traits, the range of variation of cultivars was within that of natural populations, but was close to one of the extremes of the natural populations range for some traits (Table S7).

**Figure 2:**
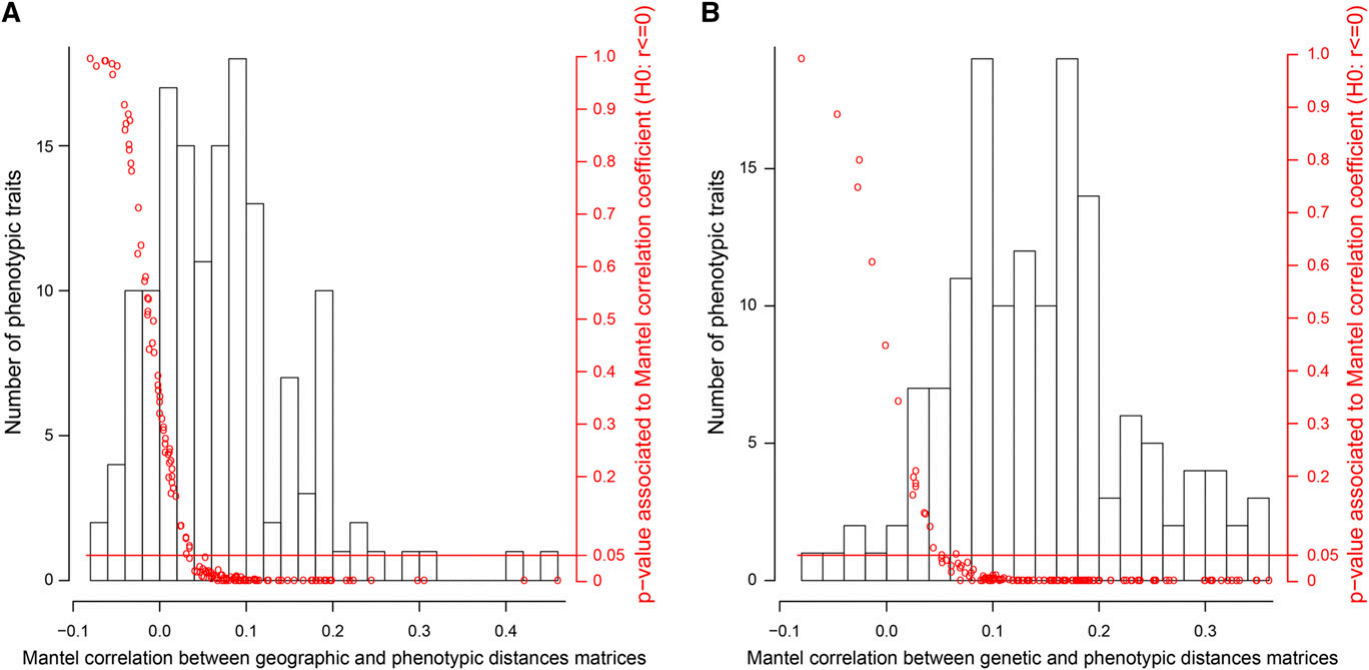
Histograms for all available traits of the Mantel correlation values between phenotypic and geographic Euclidian distances and between phenotypic and genetic Euclidian distances. Genetic Euclidian distances were computed using the allele frequencies of all available SNP markers. The red dots give the one-tailed p-value associated to each Mantel correlation value. The red horizontal line indicates the 5% p-value threshold below which the traits represented by the dots are considered to have phenotypic pairwise distances significantly correlated to geographic or genetic pairwise distances.

Pairwise phenotypic distances between natural populations were significantly positively correlated to pairwise geographic and genetic distances for over half the traits (Figure 2). The phenotypic distance between populations was generally small for small genetic distances but could vary from small to large distance for large genetic distances (Figure S4). Thus, the same phenotype can be achieved via different allele combinations.

Pearson correlation coefficients between traits are displayed in Table S8. Some important correlation patterns are reported hereafter. Many traits measured in spring such as canopy height (CHs500), protein content (*PRT_04_me17*), lamina width (*LMW_avg*) or flowering stem density (*DST_avg*) were strongly correlated (|r|>0.6) with heading date (*HEA_avg*). Vigor after cutting (*VAC_avg*) did not show correlation with heading date (r = 0) but was strongly correlated to various measures of spring growth which accounted for heading date differences (*resCHs300*, *resCHs500*) and autumn growth (*AGR*) at different environments. Autumn growth rate at PO in 2017 (*AGR_po17*) was negatively correlated to the aftermath heading score (*AHD_po17*) of the same year (r=-0.45), in agreement with an assumed cost of reproductive investment on vegetative growth. Measures of spring growth accounting for heading date in the exceptionally cold conditions of spring at PO in 2016 were poorly correlated to those in other environments (r < 0.35), but were strongly negatively correlated to the winter damage score (*WID_po16*) recorded in early March 2016 in PO (r=-0.59).

### Genetic diversity

The 189,781 SNP markers used for this study were distributed across 10,335 scaffolds (published by Byrne *et al.* 2015). A high degree of polymorphism was observed with on average one SNP every 20 base pairs in the scaffolds. However, the minor allele was rare for most SNP markers (Figure S1).

Correlation between allele frequencies of SNP markers on the same scaffold as a function of distance between SNP markers in base pairs (bp) is displayed in Figure S2. The average distance between SNP allele frequencies whose correlation was greater than 0.5, 0.25 and 0.1 was 5,351 bp, 13,397 bp and 23,603 bp respectively. The average correlation between SNP allele frequencies whose distance was less than or equal to 1,000 bp, 100 bp and 1 bp was 0.1, 0.15 and 0.24, respectively.

The average kinship between populations was close to zero, with 0.49 as the highest value between two populations. The values for the diagonal varied between 0.1 and 0.97 with 50% of the values between 0.23 and 0.42.

### GWAS results

The quantile – quantile plots (Figure S3) show that the distribution of observed p-values from GWAS models in which kinship was not accounted for, or in which only structure was accounted for, strongly departed from the expected distribution under a null model. This indicated that significance tests using such models were highly inflated and that there was a high level of false positives. This trend was removed by accounting for kinship and applying a q-value threshold. Accounting for both kinship and structure in GWAS models did not reduce the number of false positives as compared to only accounting for kinship. Therefore, we only present results from GWAS models which accounted for kinship only in the next paragraphs.

**Figure 3:**
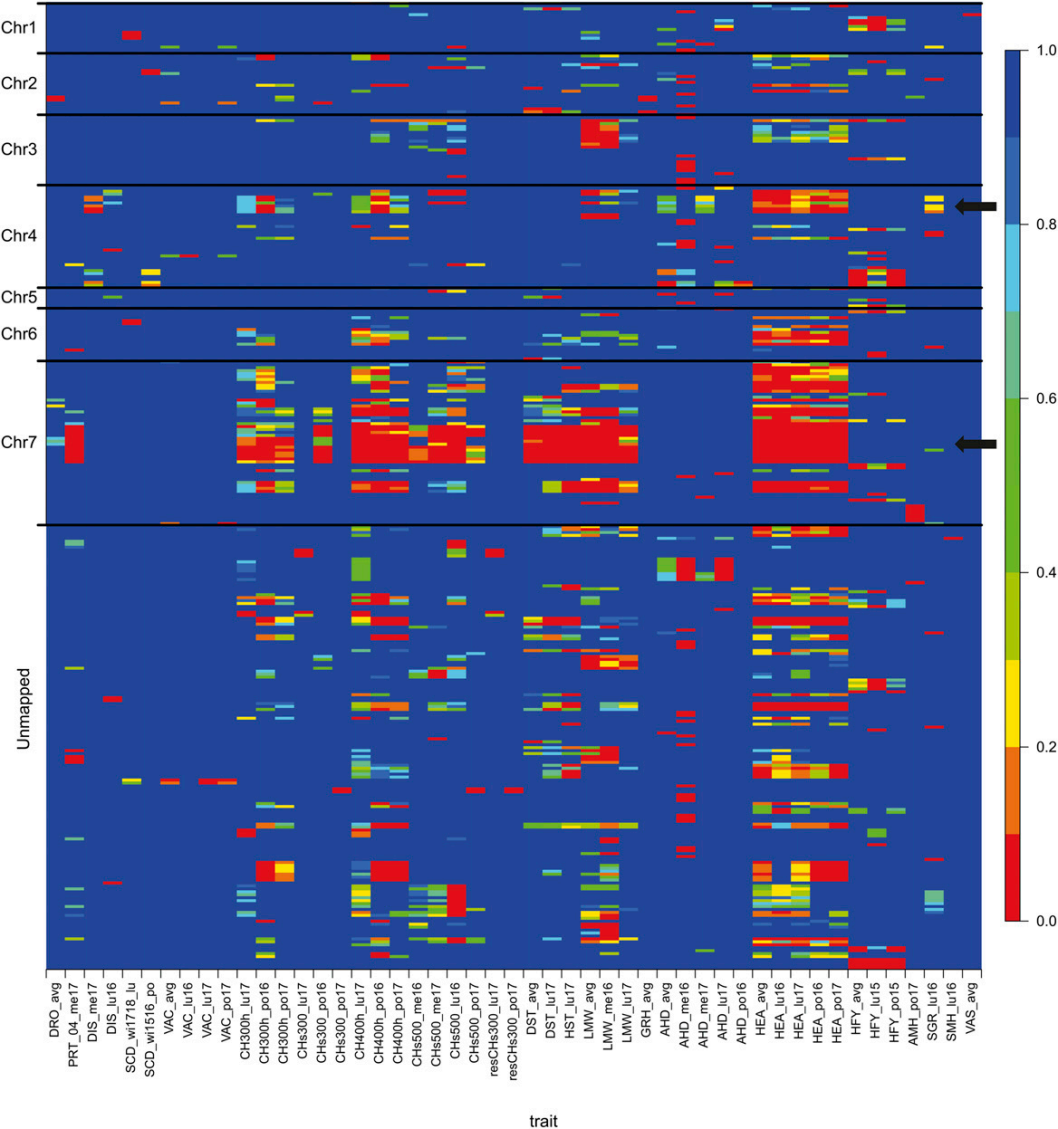
Heat map representing the q-value of associations between traits and SNP markers with the significant SNPs positioned along the different chromosomes (Chr1-Chr7 and unmapped from Byrne *et al.* 2015). The traits on the abscissa axis are those with which at least one SNP marker was significantly associated with a 10% q-value threshold and the positioned SNP markers are those associated with at least one trait (q-value < 10%). The two black arrows designate the remarkable regions on chromosome 4 and 7.

With a q-value of 10%, a total of 329 significant SNP markers distributed over 180 scaffolds were detected as associated to at least one trait (Table S9). Out of those, 226, 156 and 14 were unlinked to any other marker at correlation thresholds of 0.9, 0.5 and 0.1, respectively. One or more significant marker-trait associations were detected for 49 of the 145 phenotypic variables (Figure 3). Two remarkable scaffolds on chromosomes 7 and 4 were associated to several traits (Figure 3), notably heading date (*HEA_avg*), spring canopy height measures and lamina width (*LMW_avg*).

**Figure 4:**
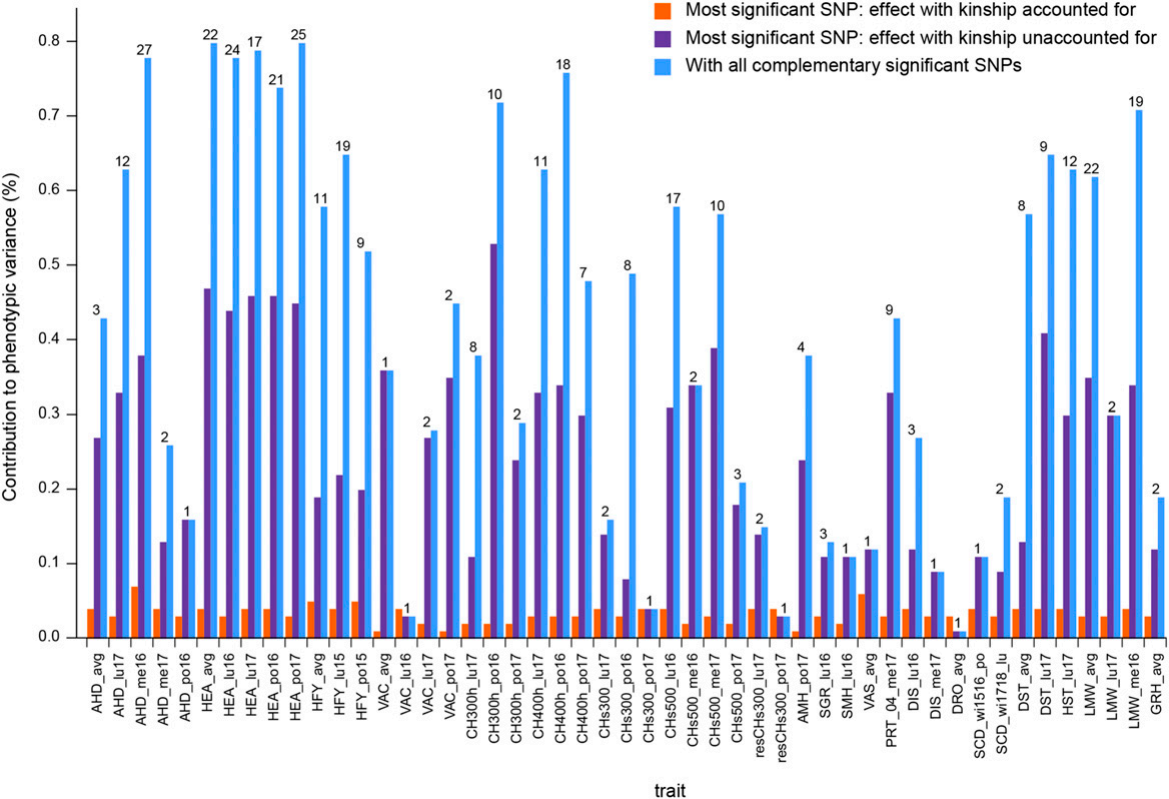
Contribution to phenotypic variance of the most significant SNPs detected by GWAS (which accounted for kinship) computed using either a model that accounted for kinship (red bars) or a model which did not account for kinship (green bars). The contribution to phenotypic variance of all complementary significant SNP detected by GWAS (q-value <10%) is also displayed (blue bars). The information is only given for traits for which a least one significant SNP was detected by GWAS (q-value < 10%). The numeric values above the blue bars indicate how many complementary SNP (each significantly improve the multiple regression model) were detected for the given trait.

The single most significant SNP marker from GWAS which accounted for kinship could explain up to 53% of the phenotypic variance (for canopy height at 300 degree days into spring growth at PO in 2016: *CH300h_po16)* if kinship was not accounted for in computing the explained variance and up to 7% of the phenotypic variance (for aftermath heading at ME in 2016: *AHD_me16*) if kinship was accounted for (Figure 4). The set of non-redundant SNP markers selected by the forward stepwise multiple regression model could explain up to 80% of phenotypic variance of a given trait (Figure 4). The number of significant SNP markers shared by pairs of phenotypic variables is presented in Table S10.

### Effect of genotype × environment interactions on GWAS results

**Figure 5:**
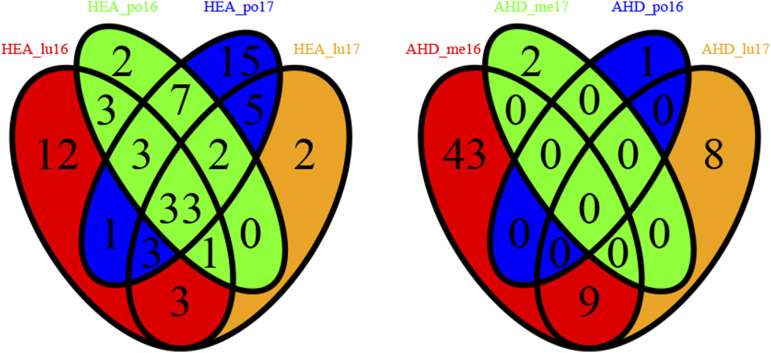
Venn diagrams displaying the number of significant SNP markers detected by GWAS (q-value < 10%) in different environments for a same trait, heading date (HEA) on the left and aftermath heading (AHD) on the right.

**Figure 6:**
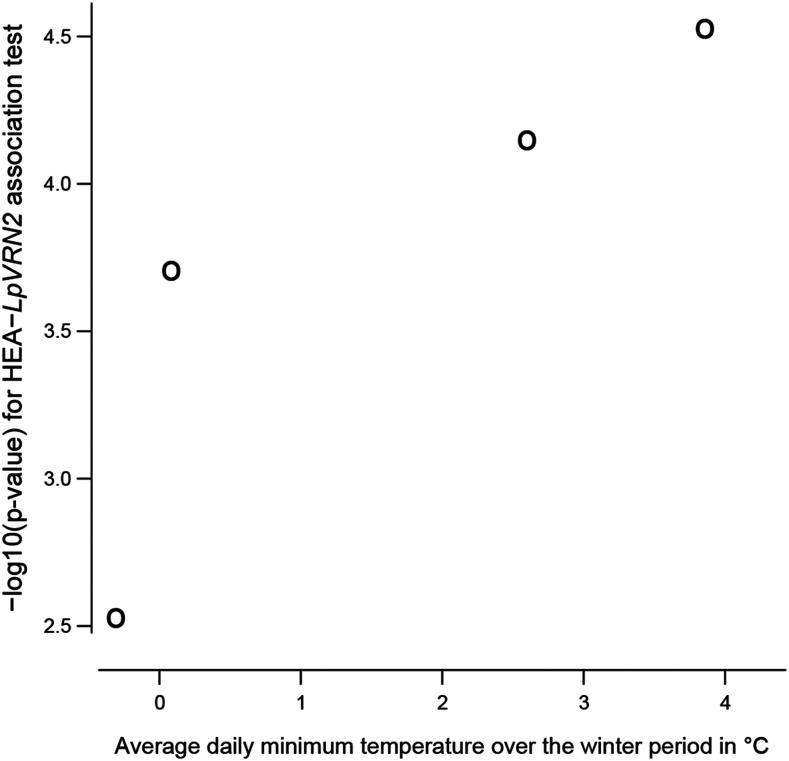
Scatterplot of the significance (-log10(pvalue)) of the association test between heading date measured in different environments and a SNP marker (position 39260 in scaffold 1700_ref0031287) located within the *LpVRN2* vernalization response gene against the average daily minimum temperature (in °C) over the preceding winter period in the environment where heading date was recorded. Environments ranked from coldest to warmest average daily minimum temperature are the following: PO-2016, PO-2017, LU-2017, and LU-2016.

For a given trait, more or less different sets of SNP markers were detected depending on the environment in which the trait was measured (Table S10) as illustrated in Figure 5 for two contrasted situations. No single SNP marker was detected as significant in all the environments in which *AHD* was recorded. In contrast, 33 SNP markers were detected as significantly linked to heading date (*HEA*) in all the environments in which it was recorded (Figure 5). However, even for this highly heritable trait, some SNP markers were significant in only one, two or three of the environmental conditions (Figure 5). Notably, the significance of the effect on heading date of a SNP marker located in the *LpVRN2* vernalization response gene (position 39260 in scaffold 1700_ref0031287) seemed to depend on the average daily minimum temperature in the trial location during the winter period preceding the heading date record (Figure 6). It was actually significant with a q-value < 10% only at LU in 2016.

### Genomic predictions

**Figure 7:**
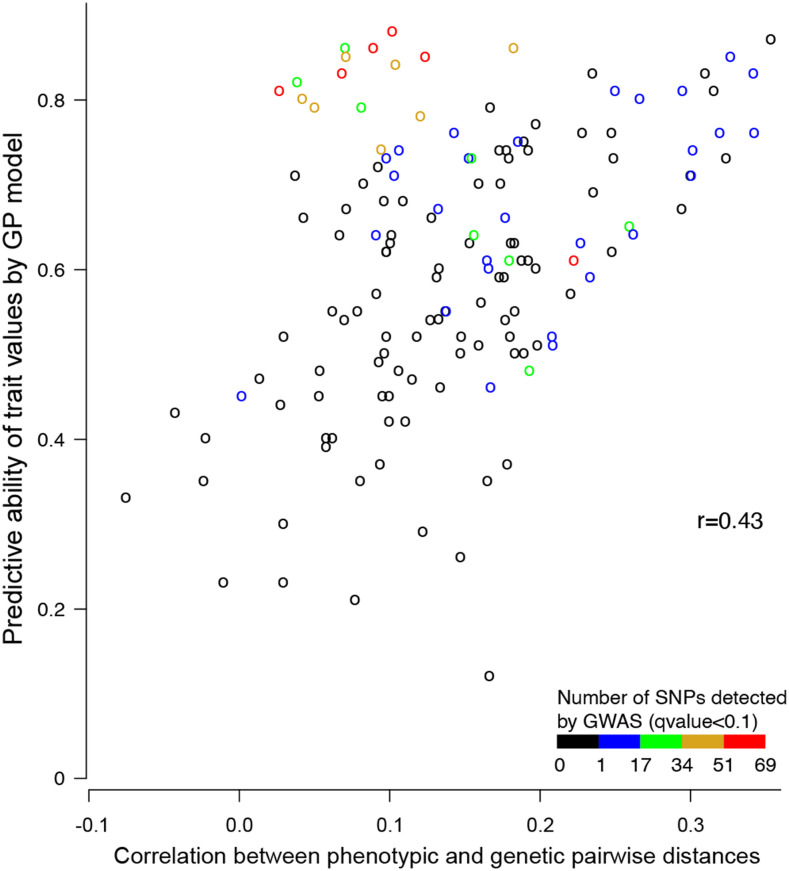
Scatterplot of the predictive ability of the genomic prediction model against the correlation between pairwise phenotypic and genetic Euclidian distances computed using all available SNP. Each dot represents a trait. The dots are colored according to the number of SNP markers detected as being significantly (considering a qvalue threshold of 0.1) linked to the trait according to a Genome Wide Association Study (GWAS). The value of the Pearson correlation between the two presented variables is given in the upper left corner. The genomic prediction models included all available SNP markers. To evaluate the predictive ability, 100 random calibration sets of all but 50 natural populations were used to predict the 50 remaining natural populations.

Predictive abilities observed in this study varied from 0.23 for regularity after sowing (*RAS*) at PO (H^2^ = 0.42) to 0.88 for heading date (*HEA*) at PO in 2017 (H^2^ = 0.98). Prediction abilities were greater than 0.5 for three quarters of the 146 variables (Table S7). Over traits, the Pearson correlation between the predictive ability and its standard deviation across the 100 samples equalled -0.75, indicating that traits accurately predicted were also less dependent on the choice of the calibration set. Among the studied traits, the predictive ability increased with the value of the correlation between genetic and phenotypic pairwise distances (Figure 7). However, for traits for which a relatively high number of markers were detected as significant with the GWAS, the predictive ability could be quite high even if the correlation between genetic and phenotypic distances was relatively small (Figure 7).

The ability of GP models calibrated on natural populations to predict the phenotype of cultivars depends on the considered trait (Table S7). The GP models were able to accurately predict cultivar phenotypes (predictive ability > 0.6) for 56 of the 145 traits, including heading date (*HEA_avg*), aftermath heading (*AHD_avg*), first year heading (*HFY_AVG*), spring growth (*CHs300_lu16*), vigor after cutting (*VAC_avg*) or disease susceptibility (*DIS_avg*) (Table S7). However, for 58 other traits such as autumn growth or forage quality traits, GP models failed to accurately predict cultivar phenotypes (predictive ability < 0.4). For traits for which more than 5 SNPs were detected by GWAS, the phenotypes of cultivars was well predicted (prediction ability greater than 0.6) by GP models calibrated using the natural populations (Table S7).

### Optimization of genomic prediction models

**Figure 8:**
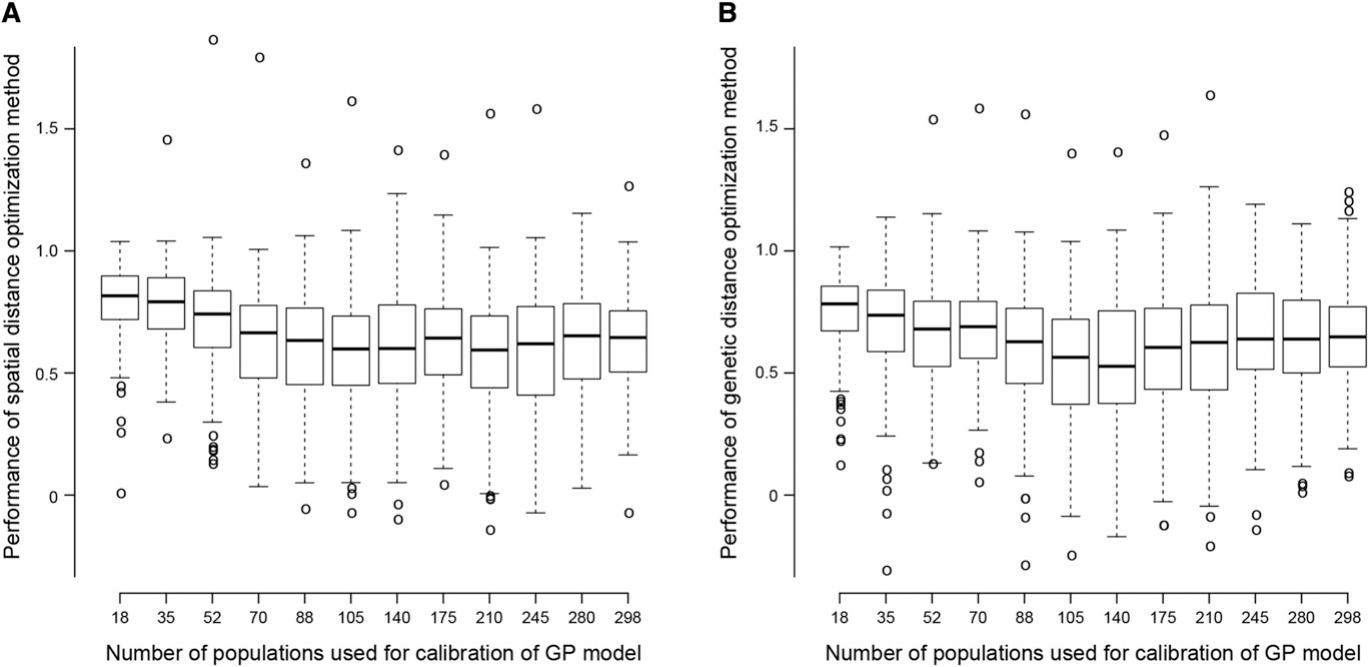
Performance of the (a) spatial and (b) genetic distances optimization methods for choosing the populations to be included in the calibration set to build a genomic prediction model against the number of populations included in the calibration set. The boxplots represent the variability of the performance between the different traits. In order to calculate the performance of an optimized calibration set, the minimum (MinPA) and maximum (MaxPA) predictive abilities over 100 randomly selected calibration sets of same size were computed. The performance of an optimized calibration set was then calculated as $\frac{\text{OptPA}-\text{MinPA}}{\text{MaxPA}-\text{MinPA}}$ with OptPA being the predictive ability obtained with the optimized calibration set. All available SNP markers were included in prediction models.

The effect on a GP model’s predictive ability of modifying the number of population included in the calibration set was evaluated. For all traits, the predictive ability of the GP model increased as the number of populations included in the calibration set increased. Indeed the correlations between the mean, minimum and maximum predictive ability across 100 different randomly sampled calibration sets and the size of the sets equalled 0.79, 0.69 and 0.83 respectively. The gain in predictive ability however quickly flattened as the size of the calibration set increased. Furthermore, there was a wide variation in predictive ability over 100 different randomly sampled sets. The average over all calibration set sizes of the standard deviation of the predictive ability across random sets equalled 0.05 on average across all traits. It varied from 0.01 for *AMH_po17* (autumn maximum canopy height) to 0.1 for *WSC_04_me17* (spring water soluble carbohydrates content). As such, the accuracy of a GP model was highly dependent on the choice of populations used to calibrate it. Several procedures were implemented to optimize this choice. Genetic and spatial optimization of the calibration set tended to perform similarly (Figure 8). However, their relative performance depended on the trait considered as shown by the average performance of optimized calibration sets over all calibration set sizes displayed in Table S7 for the different traits. The genetic optimization performance averaged over all calibration set sizes varied from 0.33 for summer canopy height at LU in 2016 (*SMH_lu16)* to above 1 for soil coverage variation during the summer of 2017 at LU (*SCD_su17_lu)* and was greater than 0.5 for 90% of the traits. The performance of optimized calibration sets was expressed relatively to a random choice of the calibration set. As such, it was generally greatest when the size of the calibration set was small (Figure 8), as a random choice led to poorly representative calibration set and low predictive ability in this case.

**Figure 9:**
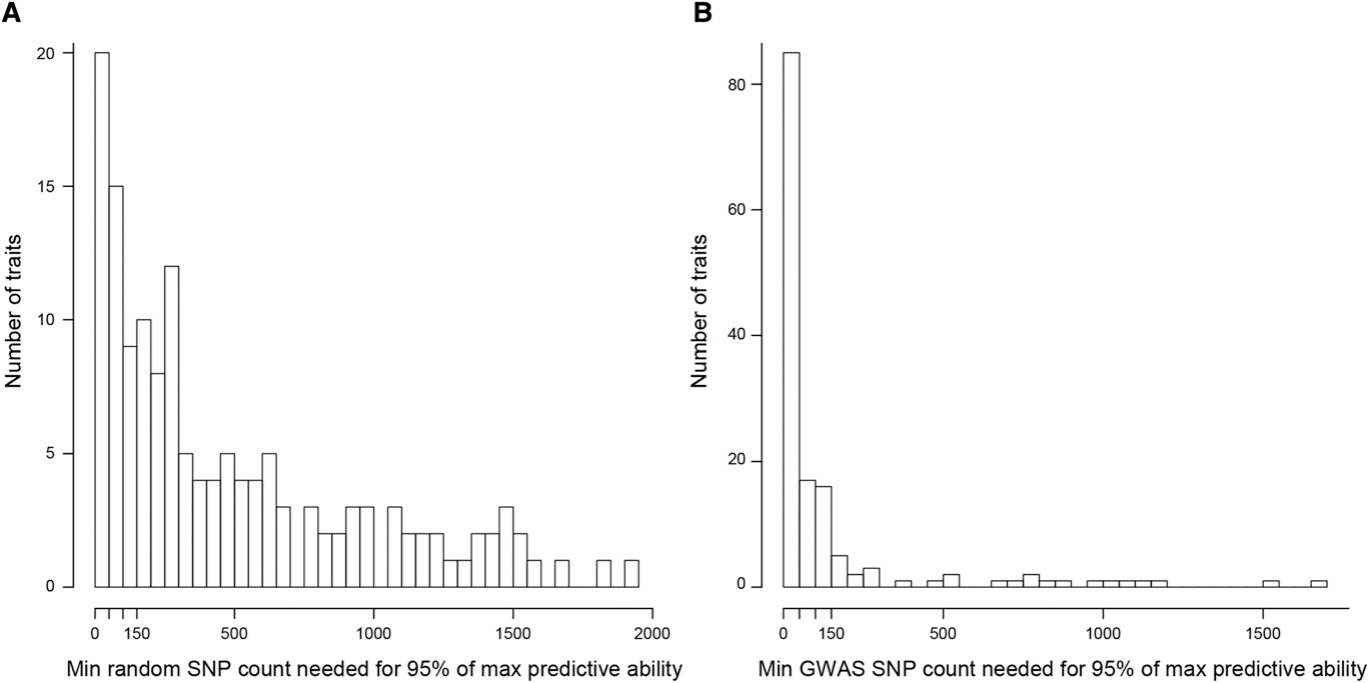
Histogram of the number of traits for which the minimum number of SNPs required to reach 95% of the maximum predictive ability of the best genomic prediction (using any number of SNPs) falls within the shown range. To reach the 95% threshold, the SNPs were either (a) randomly chosen or (b) chosen from the most to the least significant one among a non-redundant set detected in a preliminary GWAS using the calibration set of populations. The calibration set of all minus 50 populations was selected by optimizing genetic distances and the 50 populations left were used to test the models.

For all traits, the predictive ability of GP models increased as the number of used makers increased, until a threshold number above which any extra SNP marker did not provide additional improvement; this is illustrated by the minimum number of SNPs required to meet 95% of the maximum predictive ability in Figure 9 and Table S7. That threshold number varied according to traits, from 1 SNP marker for canopy height accounting for heading date at LU in 2017 (*resCHs300_lu17*) to 1,663 SNP markers for water soluble carbohydrates content in spring at ME in 2017 (*WSC_04_me17*). It was less than 200 SNP markers for 80% of the traits, notably for those for which significant (q-value < 10%) SNP markers were detected by GWAS (Table S7). For over three quarters of the traits, fewer SNP markers were required to meet 95% of the maximum predictive ability if they were not randomly drawn but rather if they included SNP markers found significantly associated with the trait in a preliminary GWAS (Figure 9 and Table S7). Furthermore, for almost all traits, the prediction model providing the largest predictive ability was obtained with an optimal number of SNP markers smaller than the total number of available SNPs (Table S7).

### Effect of genetic and phenotypic spatial structure

**Figure 10:**
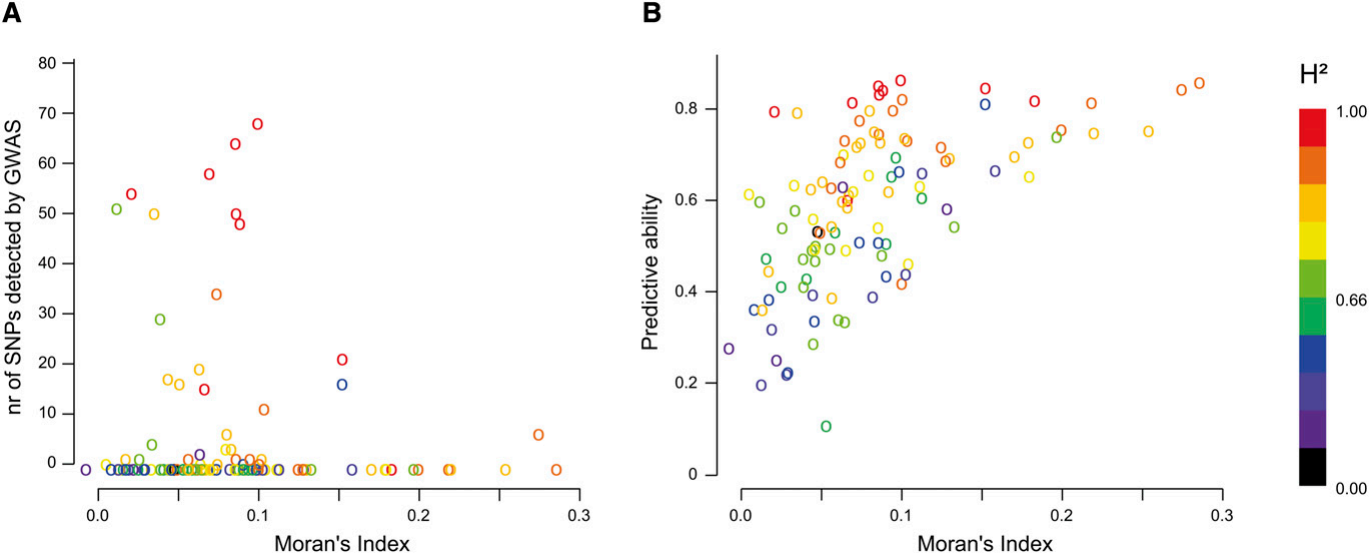
Scatterplot of Moran’s index and of (a) number of SNP markers detected by GWAS (q-value < 10%) and (b) observed predictive abilities of genomic prediction models for the different phenotypic traits analyzed in the study. Dots are colored according to the trait H^2^ (broad sense heritability like indicator).

**Figure 11:**
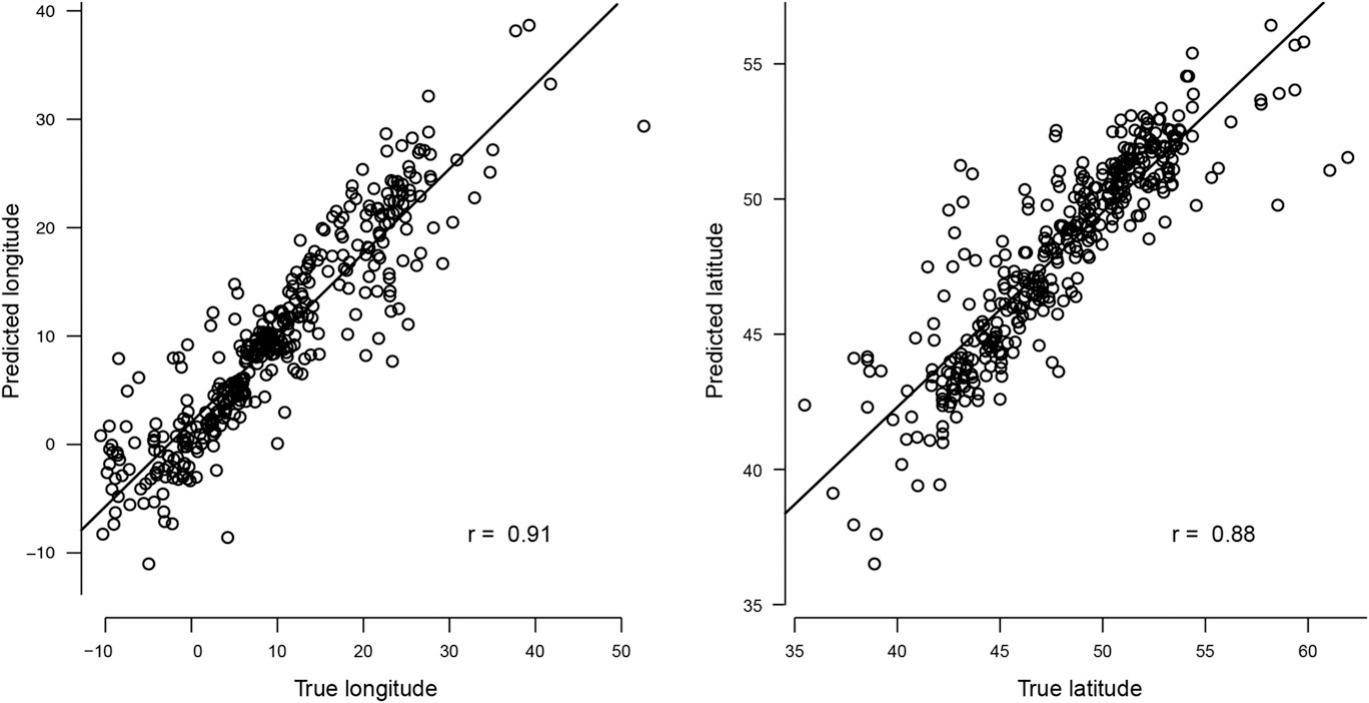
Scatterplot of predicted and true (a) longitude and (b) latitude (WGS84, DD) of sites of origin of perennial ryegrass populations. Leave one out cross validation was used to evaluate the genomic prediction models. All available SNP markers were used. Prediction abilities are assessed by r (lowest right corner).

Moran indices are presented for all traits in Table S7. For traits whose Moran’s index was higher than 0.15 (trait value highly linked to geographical origin), the GWAS detected very few SNP markers even though the heritability was high (Figure 10-a). However, the predictive ability of GP was high for all these traits (Figure 10-b). The geographical origin of natural populations could be fairly well predicted using genetic information (Figure 11), the distance between predicted and true position being less than 200 km for over half the populations. Furthermore, a correlation of 0.44 was found between genetic and geographical distances. Genetic isolation by geographic distance therefore shaped the diversity of this species. Traits whose values were highly linked to spatial origin were thus accurately predicted using kinship in GP but had few associated SNPs detected by GWAS models which accounted for genetic relatedness.

## Discussion

### Phenotypic diversity

Since grasslands need to be adapted to the changing climate while maintaining a sufficient agronomical value, we aimed to evaluate the natural genetic diversity of perennial ryegrass for traits expected to contribute to environmental adaptation and/or agronomical value. Based on literature about environmental adaptation of grass species, some traits could be presumed as adaptive. Those include autumn growth rate, reproductive tiller density, heading earliness (Barre *et al.* 2018), isotopic composition of ^13^C (Condon *et al.* 2002), vegetative plant height before stem elongation (Oyarzabal *et al.* 2007), aerial dry matter lignin content (Casler *et al.* 2002), water soluble carbohydrates content of lamina or aerial dry matter (Humphreys 1989; Volaire 1995; Sanada *et al.* 2007). In our study, different patterns of spatial autocorrelation were observed according to traits (Table S7). Those spatial autocorrelations represent genetic patterns since they were calculated using the coordinates of the sites of origin of the populations and phenotypic data recorded at common trial sites. This suggests that demographic and selection processes have not similarly impacted the genetic diversity involved in the determinism of these various traits. As it has been proposed that most of the natural phenotypic diversity is due to more or less ancient Darwinian selection (Rieseberg *et al.* 2002), most traits for which wide natural genetic diversity has been observed may be adaptive in some way.

To apprehend the services that raw natural diversity can render to farmers, traits of interest for forage production were evaluated. Those include forage quality and phenological traits, disease resistance, vigor after cutting and growth at various seasons assessed through the measurement of canopy heights. We found that canopy height was fairly well correlated (File S1) to dry matter yield (r > 0.8) recorded at the same date during the spring vegetative growth, which confirms that canopy height could be used as an indicator of biomass production (Powell 1974).

The relatively high H^2^ values observed for most traits (Table S7) indicated that great inter-population variance was revealed by the field trials implemented for this study. For example, heading date heritability was greater than 0.97 in all environments, which is higher than values reported in previous studies (Fè *et al.* 2015b; Barre *et al.* 2018). The dry matter yields measured on a sample of plots at each site (File S1) were within a common range for perennial ryegrass (McDonagh *et al.* 2016).

Population × environment interactions were highly significant for all recorded traits, even those linked to phenology such as heading date (Table S7). This could be explained by the marked differences of climate between the three experimental sites. For example, the spring growth (weekly records of canopy height) in the exceptionally cold conditions at PO in 2016 (Table S5) was poorly correlated to that observed in other locations or in other spring season at PO (Table S8). Adaptation to frost stress was likely revealed for some populations that grew best in spring 2016 at PO since they also suffered the less damage in the preceding winter (2015-16). A previous study which evaluated perennial ryegrass natural populations in experimental sites located in France also revealed strong population × environment interactions even for phenological traits such as heading date (Charmet *et al.* 1990).

Traits important for forage production often displayed different phenotypic distributions between the set of cultivars and the set of natural populations (Table S7). The values of cultivars were often tightly distributed closer toward an interesting extreme for forage production. However, for all traits, the range of values shown by the cultivars was within (or close to) the range of values shown by the natural populations. This was even the case for productivity traits such as spring canopy heights or autumn growth rates and for forage quality traits such as lignin content. A previous study on natural perennial ryegrass populations also revealed that even though natural populations showed on average lower agronomic performances, their range of variation was much larger (Balfourier and Charmet 1991). Such results indicate that natural diversity holds potential for use in breeding programs to further improve the agronomic value of cultivars as suggested by other authors (Charmet *et al.* 1989). Novel combinations of natural sources could likely generate original phenotypes including more extreme trait values and/or new combinations of trait values that would be worthwhile for both climatic adaptation and agronomical value.

### Valorising GWAS results

Population allele frequencies and mean phenotypes per population have already been successfully combined for association studies to detect major effect loci in various species such as *Drosophila melanogaster* (Bastide *et al.* 2013), humans (Gaj *et al.* 2012; Riaz *et al.* 2016) and perennial ryegrass (Fè *et al.* 2015a). This was also the case in the present study in which areas of the genome including causal genes for various traits were revealed (Table S9).

A notable example is a region on chromosome 7 which appeared linked to heading date as well as spring growth, leaf lamina width, spring forage quality traits, flowering stem density and straw height (Figure 3). Another region on chromosome 4 appeared linked to first year heading, heading date and aftermath heading in multiple environments. One hypothesis is that such regions of the genome contain pleiotropic genes (Stearns 2010) meaning that all associated traits would have a common genetic determinism. Another hypothesis is that such regions contain multiple genes in linkage (Zeven and Harten 1979) that would correspond to co-adapted functions and for which limited allelic arrangements provide high adaptive values.

The number of QTL detected by GWAS and the percentage of phenotypic variance explained varied greatly between traits (Figure 4 and Table S9). The effect of a single SNP on phenotypic variation in a GWAS model which accounted for kinship was likely underestimated due to the strong confusion between the genetic background and the distribution of values for most traits. As such the effect of each SNP detected by GWAS (which accounted for kinship) was also computed using a linear regression model between trait values and SNP allele frequencies which did not account for kinship. That method on the contrary likely overestimated the SNP effect. The common GWAS issue of missing heritability (Manolio *et al.* 2009) was more or less important depending on the trait. In no case could the SNP markers explain all of the inter-population genetic variance.

The four following reasons could explain the poor capacity of GWAS to detect significant makers for a number of traits. (i) Even if the number of markers used was high, some QTL could have been missed due to the fast LD decay. (ii) Because of confusion between genetic kinship and phenotypic similarity, accounting for kinship in GWAS models could have hampered the detection of QTL. For natural diversity in which diversifying selection and gene flow are strong, it has been proposed that only a small number of large effect QTL that shape inter-population trait variability may show high levels of allelic differentiation (Corre and Kremer 2003; Storz 2005; Bell 2010). A large portion of the loci involved in the variability of a trait may behave like neutral markers in terms of allelic differentiation. Consequently, GWAS models accounting for kinship could only detect large effect adaptive QTL. Furthermore, the continuous genetic structure due to isolation by distance has for consequence that few or no significant SNPs could be detected by GWAS for traits showing high spatial autocorrelation. (iii) A high proportion of the GBS SNP markers had a rare minor allele (present in few populations and/or at low frequency in populations). As reported from previous studies (Sanchez-Bermejo *et al.* 2015), rare alleles may have been responsible for a substantial part of the phenotypic variation of many traits and make their genetic determinism difficult to apprehend. (iv) Another hypothesis is that the variation of some traits results from many genes with small effects. It was suggested (Nicolae *et al.* 2010; Zhao *et al.* 2011) that the integration of transcriptome data could improve the ability to detect moderate effect loci if gene expression levels can be associated to phenotypic variation, *i.e.*, in the case of expression quantitative trait loci (eQTL).

For all traits, there are cases of phenotypically similar populations that are spatially and genetically distantly related even if genetic distance is calculated using the allele frequencies of the SNPs most significantly associated to the trait (Figure S4). This suggests that the causal polymorphism can vary according to geographical regions. This shows that the search for natural genetic resources to improve a given trait, or adaptation to a given climatic constraint, should generally not focus on a single restricted geographical region. Original genotypes recombining valuable alleles at various loci could be designed through transgressive segregation (Rieseberg *et al.* 1999).

Population × environment (G×E) interactions should be taken into account when implementing GWAS. Many phenotype-SNP associations were environment-dependent (Table S10), a likely common issue in association studies that is often ignored (Hirschhorn *et al.* 2002). The effect of environment on biological systems is multifactorial and it is thus difficult to determine which environmental factor, or combination of factors, interacts with the QTL. Therefore, it may be difficult to predict the effect of environment interactive SNPs in a new environment and so to use such markers reliably. However, when a SNP marker is located within a gene whose function is known, assumptions may be formulated to explain SNP × environment interactions. For example in this study, the effect of a SNP in the *LpVRN2* vernalisation gene (Andersen *et al.* 2006) varied according to year and location (Figure 6) as if cold winters were less likely to lead to differences in heading date determined by variation in vernalization response. To confirm such relations and to further investigate GxE interactive traits, it may be necessary to perform association studies using the reaction norm slope of a given trait to an environmental variable obtained across many different environments (Sanchez-Bermejo *et al.* 2015) .

### Genomic predictions

The results of this study are very promising as to the ability of GP models to accurately predict the value of natural populations of a grassland species for many traits relevant to agronomical value and/or environmental adaptation (Figure 7, Table S7). GP were already tested on various forage species notably alfalfa for which Jia *et al.* (2018) found the highest accuracy (0.65) for autumn plant height, switchgrass for which Ramstein *et al.* (2016) did not find accuracies exceeding 0.5 and perennial ryegrass for which results are very variable as described in the following. Faville *et al.* (2018) reported a study in which 517 mother plants were sampled from a commercial breeding program, genotyped for over one million SNP markers and their half-sib progeny families were phenotyped. That study led to predictive abilities that did not exceed 0.52 for heading date. Pembleton *et al.* (2018) reported a study based on 18 years of phenotypic data recorded in a commercial breeding program involving 714 synthetic populations that were genotyped for over 28,000 SNP markers. They reported a predictive accuracy of 0.31 for biomass yield and 0.76 for heading date. Other than the relatively high number of available SNP markers and phenotyped accessions, what distinguishes our study is both the use of allele frequencies of populations rather than individual genotypes and the use of natural highly diverse genetic material.

More or less ancient selection may have been the main driving force that shaped the extant phenotypic variability of natural populations (Rieseberg *et al.* 2002). The natural populations we studied originated from contrasting environments likely requiring different local adaptive trait optima and imposing different selection pressures at the underlying QTL. These QTL should thus have outstanding allelic distributions as compared to neutral loci affected mainly by drift and migration. Yet, due to the outbreeding nature of perennial ryegrass, gene flow between spatially close populations is likely fairly strong, as suggested by the correlation of 0.44 between geographic and genetic distances which suggests isolation by distance. As such, it is likely that high levels of allele differentiation from the genome-wide genetic structure only exist for the few largest effect QTL under strong selection (Corre and Kremer 2003; Bell 2010) . The allele distributions of most loci involved in the phenotypic variability of adaptive traits are likely confounded with the neutral continuous genetic structure. For non-adaptive traits, the distribution of population values and of allele frequencies at most of the underlying QTL is expected to follow neutral patterns and to be more specifically ruled by isolation by distance. As such, for most traits the patterns of isolation by distance for the phenotypic values and for the genetic background are comparable, thus making kinship analyses (genetic relatedness) a very powerful tool for predicting phenotypic differences at the ‘species scale’. The high predictive abilities obtained for phenotypic traits could be largely thanks to a good estimation of kinship with high density molecular markers.

The preceding hypothesis could explain the differences of predictive ability between natural populations and cultivars (Table S7), which could be related to the genetic architecture of predicted traits. For example, heading date is thought to be under the control of few large effect genes (Fè *et al.* 2015a) as our GWAS results confirmed, whereas autumn growth appeared more likely to be under the control of many small effect genes. It could thus be that heading date was well predicted both for natural populations and cultivars because the prediction model uses information linked to loci that have a large and stable effect regardless of the genetic background. Conversely, it could be that autumn growth, which shows strong spatial autocorrelation, was well predicted for natural populations largely because of a good estimation of kinship but the models were far less effective for the cultivars for which the genetic structure has been broken by genetic recombination cycles.

The use of GP models to describe genebank accessions was already evaluated for various domesticated plant species such as wheat (Crossa *et al.* 2016), sorghum (Yu *et al.* 2016), cauliflower (Thorwarth *et al.* 2018), pea (Burstin *et al.* 2015), soybean (Peixoto *et al.* 2017) and for the natural diversity of *Arabidopsis* (Kooke *et al.* 2016). In most of these studies, as well as in ours, predictive abilities were remarkably high for many traits and genetic structure (continuous or stratified) seemed to play an important role. For example, Yu *et al.* (2016) reported an accuracy of 0.84 when predicting dry biomass yield for 200 accessions of sorghum with a model that used over 250,000 SNP markers. However, the high accuracy appeared to be mainly due to the ability of the model to reveal phenotypic differences between genetic groups and not so much within groups. Population structure is expected to be high in genebank collections capturing large portions of intra-specific variability compared to that within commercial breeding material (Crossa *et al.* 2016). Such structure could partly explain the high predictive abilities observed in the above quoted studies in which models likely used kinship to predict breeding values of traits whose distribution was more or less confounded with genetic structure. Here, we argued that the continuous genetic structure in our genetic material, as previously reported by Blanco-Pastor *et al.* (2019), likely explains the high predictive abilities we obtained.

### Practical use of GP for mining natural genetic resources

Describing a non-phenotyped portion of genetic resources by the mean of GP models calibrated with phenotypic data recorded on a another portion of these genetic resources leads to query about which resources should be phenotyped and what genotyping effort should be made.

Optimization of calibration sets for GP models, based on stratified sampling was previously shown to be effective (Isidro *et al.* 2015). The methods used in the present study were similar except that the size of the desired calibration set equals the number of clusters to choose from. If all populations or individuals from a genetic resource collection are genotyped, our results show that genetic distance optimization can be successfully implemented. However, if genotyping information is not available, our results show that it can be efficiently substituted by geographical information if phenotypic diversity is spatially structured. Stratified sampling in spatial clusters was already shown to be an efficient way to choose a core collection of natural populations of perennial ryegrass (Balfourier *et al.* 1998). The best choice between optimization based on genetic or geographical information depends on the trait and the relative size of the calibration set. The use of stratified sampling after clustering based on both genetic similarity and spatial proximity appeared to be the most stable (across traits and calibration set sizes) procedure to choose a calibration set. Such a procedure makes possible the choice of a single subset of accessions that can be used to calibrate prediction models for any trait without prior knowledge of their heritability. This is a benefit compared to method such as CDmean optimization (Rincent *et al.* 2012) which gave us similar results in terms of predictive ability gains relative to random sampling (not shown) but must be applied trait by trait.

Regarding the genotyping effort, it could be considered to optimize the number and choice of SNP markers to use. Many previous studies showed, as we did, that the predictive ability of genomic models improves as the number of included SNP markers increases until a saturation is reached (Spindel *et al.* 2016; Thorwarth *et al.* 2018). For any trait, 1000 random SNPs appears sufficient to reach near maximal predictive abilities, which suggests that the estimation of kinship does not indefinitely improve with the addition of extra genetic information. Furthermore, the GP of many traits was improved by including markers identified as most significant in a GWAS based on the calibration set data. Such markers may not necessarily be linked to QTL but may simply have an allele whose population frequency distribution is particularly well correlated to the phenotypic diversity for the trait considered.

In the present study, the populations were sampled across the natural geographic range of perennial ryegrass so that most of the genetic diversity of the species may be represented. Hence, if GP models calibrated on a sub-set of the sampled populations can accurately predict the remaining populations, they should also accurately predict any other natural genetic material already present in a genebank or collected in its native environment. Consequently, this study shows that GP can greatly improve capabilities to mine the whole natural genetic diversity of a species for many traits of agronomical and/or ecological importance. Furthermore, the objective of choosing an optimized set of populations for the calibration of GP models, as presented in this study, is quite similar to the objective of choosing an optimized core collection representative of the genetic diversity to be safeguarded in a genebank. Setting up such core collections is relevant to maximize the ratio of preserved diversity to conservation costs but also to study the diversity of a species with a representative sample of limited size (van Hintum *et al.* 2000).
